# Supplementary material for: RIPK1 is essential for Herpes Simplex Virus-triggered ZBP1-dependent necroptosis in human cells
Source: bioRxiv. 2024 Sep 19:2024.09.17.613393. Preprint. [Version 1] doi: 10.1101/2024.09.17.613393 (PMC11429907; doi:10.1101/2024.09.17.613393)
Supplement: 1 [file NIHPP2024.09.17.613393v1-supplement-1.pdf]

bioRxiv preprint doi: https://doi.org/10.1101/2024.09.17.613393; this version posted September 19, 2024. The copyright holder for this preprint (which was not certified by peer review) is the author/funder, who has granted bioRxiv a license to display the preprint in perpetuity. It is made available under aCC-BY 4.0 International license.

SUPPLEMENTAL LEGENDS

Figure S1. Related to Figures 2 and 3. (A-F) Cell death kinetics of SVEC4-10 (A), 3T3-SA (C), and MEF-mZBP1 (E) cells treated with DMSO or R1-ICR-3 (1μM) for 5 hrs, followed by HSV-1(ICP6mut) infection. Immunoblotting of SVEC4-10 (B), 3T3-SA (D), and MEF-mZBP1 (F) cells pretreated with DMSO or R1-ICR-3 (1μM) for 5 hrs, followed by HSV-1(ICP6mut) infection and subjected to immunoblotting with p-MLKL, MLKL, RIPK1, ICP0, and GAPDH antibodies. (G) Cell death kinetics of MEF-mZBP1, followed by HSV-1(ICP6mut) infection, with or without Nec-1, GSK963, GSK872, NSA, or zVAD. (H) MEF reconstituted with empty vector (EV) or Flag-mZBP1, were either mocked or infected with HSV-1(ICP6mut) for 10 hrs. Co-immunoprecipitation was performed in these cells. Results are representative of at least two independent experiments. Error bars represent mean ± SD.

Figure S2. Related to Figure 5. (A) Cell viability of MEFs reconstituted with the indicated mouse ZBP1 constructs, followed by HSV-1(ICP6mut) infection. Viability was determined at 18 hpi by CellTiter-Glo assay. (B) Cell death kinetics of MEFs reconstituted with mZBP1 mutants, followed by HSV-1(ICP6mut) infection. (C) The expression levels of mZBP1 in MEFs reconstituted with mZBP1 constructs were confirmed by immunoblotting with Flag and GAPDH antibodies. One-way ANOVA and Dunnett's multiple comparisons tests were used to test for statistical differences in (A). *P < 0.1, **P < 0.0001, ***P < 0.001, ****P < 0.0001. Individual data points indicate three

bioRxiv preprint doi: https://doi.org/10.1101/2024.09.17.613393; this version posted September 19, 2024. The copyright holder for this preprint (which was not certified by peer review) is the author/funder, who has granted bioRxiv a license to display the preprint in perpetuity. It is made available under aCC-BY 4.0 International license.

technical replicates. Results are representative of at least two independent experiments. Error bars represent mean ± SD.
